# Atopic Dermatitis: Striving for Reliable Biomarkers

**DOI:** 10.3390/jcm11164639

**Published:** 2022-08-09

**Authors:** Styliani Mastraftsi, Georgia Vrioni, Michail Bakakis, Electra Nicolaidou, Dimitrios Rigopoulos, Alexander J. Stratigos, Stamatios Gregoriou

**Affiliations:** 1 1st Department of Dermatology and Venereology, Andreas Sygros Hospital for Skin and Venereal Diseases, Medical School, National and Kapodistrian University of Athens, 16121 Athens, Greece; 2 Department of Microbiology, Medical School, National and Kapodistrian University of Athens, 11527 Athens, Greece

**Keywords:** atopic dermatitis, biomarkers, precision medicine, personalized treatment, TARC/CCL17, MDC/CCL22, CTACK/CCL27, periostin, IL-13, IL-22

## Abstract

Atopic dermatitis (AD) is a highly heterogeneous inflammatory disease regarding both its pathophysiology and clinical manifestations. However, it is treated according to the “one-size-fits-all” approach, which may restrict response to treatment. Thus, there is an unmet need for the stratification of patients with AD into distinct endotypes and clinical phenotypes based on biomarkers that will contribute to the development of precision medicine in AD. The development of reliable biomarkers that may distinguish which patients with AD are most likely to benefit from specific targeted therapies is a complex procedure and to date none of the identified candidate biomarkers for AD has been validated for use in routine clinical practice. Reliable biomarkers in AD are expected to improve diagnosis, evaluate disease severity, predict the course of disease, the development of comorbidities, or the therapeutic response, resulting in effective and personalized treatment of AD. Among the studied AD potential biomarkers, thymus and activation-regulated chemokine/C-C motif ligand 17 (TARC/CCL17) has the greatest evidence-based support for becoming a reliable biomarker in AD correlated with disease severity in both children and adults. In this review, we present the most prominent candidate biomarkers in AD and their suggested use.

## 1. Introduction

Atopic dermatitis (AD) is a common inflammatory skin disorder affecting up to 25% of children and up to 10% of adults [1,2]. Apart from the inflammation of the skin, AD is also characterized by systemic inflammation manifesting with other atopic (asthma, allergic rhinitis), non-atopic (cutaneous lymphomas) and psychiatric (anxiety, depression) co-morbidities, epidermal barrier dysfunction and persistent pruritus [3]. AD is often associated with elevated serum immunoglobulin E (IgE) levels and a personal or family history of type I allergies. Its course involves chronic relapses that significantly compromise the patients’ quality of life [1]. With its very high incidence in childhood, chronicity, devastating effect on quality of life for the affected patients and their families, enormous socioeconomic costs, and the recent development of new promising therapeutic options, AD represents a major challenge.

AD has a complex pathogenesis involving multiple genetic, immunologic, and environmental factors, which leads to a dysfunctional skin barrier and dysregulation of the immune system [4]. The disease is characterized by high heterogeneity in both pathophysiology and clinical manifestations leading to its classification into different endotypes or subtypes and clinical phenotypes [5]. Regarding immunologic heterogeneity, although AD is primarily characterized by excessive activation of type 2 T helper (Th2) cells and type 2 innate lymphoid (ILC2) cells, with a predominant increase in type 2 cytokines in the skin, the additional activation of Th1, Th17 and Th22 cytokine pathways also contributes to its pathogenesis. [6,7]. Moreover, the clinical manifestations of AD can be classified according to their correlation with patients’ age, disease severity, age of onset and ethnic origin [8].

Despite the high degree of clinical and molecular heterogeneity of AD, the disease is currently treated according to the “one-size-fits-all” approach, which may restrict the efficacy of the therapy administered [8]. Even the newly developed biologic agents that target specific cytokines or their receptors and the broad-acting Janus kinase inhibitors fail to completely control disease in most AD patients [9,10,11,12]. In addition, to date, the diagnosis is exclusively based on clinical criteria due to the lack of distinct laboratory or histological features, while the quantification of disease severity using measurement tools, such as the SCORing of Atopic Dermatitis (SCORAD), Eczema Area and Severity Index (EASI) and Investigator’s Global Assessment (IGA), relies on the observer’s subjective assessment [13]. Therefore, there is still a high unmet medical need for the identification of reliable biomarkers that could reduce observatory differences and would be useful as objective tools for the diagnosis, disease severity measurement and especially the development of precision medicine in AD.

Precision medicine strives to tailor health care to individual patient characteristics. These individual characteristics include information on genetics and epigenetics, health history, lifestyle and beyond, based on which individuals can be classified into subpopulations, and which appropriate preventive or therapeutic interventions can be precisely concentrated on those in need, leading to a medical care regime of higher efficiency and lower risk for individuals, and lower cost in general. Precision medicine efforts are supported by the tailwinds of cost and quality pressures of countries around the globe, holding a significant potential in the discovery, development, and application of precision prevention, diagnostics, therapeutics, and prognostics. These efforts should be at the forefront of research, particularly in countries where the potential for reimbursement of new high-cost therapies is limited. As a result, taking into consideration the multifactorial pathogenesis of AD and its heterogeneous clinical and molecular phenotypes, the stratification of AD patients into homogeneous subgroups based on the expression of accurate and reliable biomarkers is essential for effective predictive, preventive, and personalized AD management.

## 2. Definition and Subtypes of Biomarkers

To date, there are available several, although overlapping, definitions of the term biomarker. A biomarker or biological marker is defined as a “characteristic that is objectively measured and evaluated as an indicator of normal biologic processes, pathogenic processes, or pharmacologic responses to a therapeutic intervention”, according to the National Institutes of Health (NIH) [14]. Another definition of biomarker proposed by the World Health Organization (WHO) is as follows: “any substance, structure or process that can be measured in the body or its products and influence or predict the incidence of outcome or disease. Biomarkers can be classified into markers of exposure, effect, and susceptibility” [15].

Since biomarkers are widely used in the process of drug discovery, development and approval, the initial definition of NIH has evolved into a broader, enriched definition of biomarker adopted by the Food and Drug Administration (FDA) namely: “a defined characteristic that is measured as an indicator of normal biological processes, pathogenic processes, or responses to an exposure or intervention, including therapeutic interventions. Molecular, histologic, radiographic, or physiologic characteristics are types of biomarkers. A biomarker is not an assessment of how a patient feels, functions, or survives” [16]. Moreover, the European Medicines Agency (EMA) defines a biomarker more restrictively as ‘‘a biological molecule found in blood, other body fluids, or tissues that can be used to follow body processes and diseases in humans and animals’’ [17].

The biologic origin of a biomarker could be genomic information, transcriptomic profiles obtained by analysis of mRNA and miRNA, proteins such as cytokines and other mediators from body fluids (whole blood, serum, plasma, tissue fluids) or tape stripping, and morphological information [18]. Regarding the purpose/value of biomarkers, there are seven different categories as defined by the FDA-NIH Biomarker Working Group: susceptibility/risk, diagnostic, monitoring/severity, prognostic, predictive, pharmacodynamic/response, and safety [16]. Thus, evaluation of all these subtypes of biomarkers could play a significant role in the diagnosis, prognosis, management, and treatment of AD.

## 3. Biomarkers in AD

Clinical research has discovered several different subtypes of potential biomarkers in AD. However, to date none of these candidate biomarkers are used in routine clinical practice, since they have not yet reached the status of validation and qualification [19]. Recently, an international panel of experts consented that the most important performance elements for high-quality AD biomarkers are reliability, clinical validity, relevance, and high positive predictive value. Regarding the purpose of biomarkers in AD, the prediction of therapeutic response and disease progression was considered the most important. Additionally, insufficient validation by independent researchers was reported as a major obstacle to the transfer of AD biomarkers in clinical practice. All experts identified validation and further studies as a high-priority research objective [2].

Most of the skin biomarkers in AD have been identified using whole-tissue skin biopsy for sample retrieval, an invasive approach that is not always feasible, especially in the pediatric population. Consequently, less invasive sampling methods have been recently developed for AD biomarker assessment, such as the use of tape strips [20,21,22,23], dried blood spots [24], patients’ serum [25], or saliva [26].

Candidate AD biomarkers can be divided into different subtypes according to their suggested use.

### 3.1. Biomarkers for Disease Clinical Severity and Monitoring Disease Activity

Many potential biomarkers have been described in the literature correlating with AD severity and monitoring of disease activity during treatment. Since Th2 and Th22 immune pathways predominate in AD inflammatory response, these potential biomarkers include Th2- and Th22-related cytokines and chemokines identified both in skin and serum, namely, interleukin (IL)-13, IL-22, thymus and activation-regulated chemokine/C-C motif ligand 17 (TARC/CCL17), pulmonary and activation-regulated chemokine (PARC/CCL18), macrophage-derived chemokine (MDC/CCL22), cutaneous T-cell-attracting chemokine (CTACK/CCL27) and eosinophil-attracting chemokine (eotaxin-3/CCL26) [18,25]. However, according to a recent study, such AD biomarkers are mostly elevated in patients with moderate to severe and not mild disease [27].

Moreover, in another study, a combination of four serum biomarkers, namely TARC/CCL17, PARC/CCL18, IL-22 and soluble IL-2 receptor (sIL-2R), demonstrated a better correlation with disease severity compared to a single biomarker in AD patients [28]. Similarly, a combination of biomarkers involved in different immunological pathways was found to correlate better with AD severity than individual biomarkers in a few other studies conducted [25,29,30], emphasizing the multifactorial and complex pathogenesis of AD.

Nevertheless, TARC/CCL17 was found to be the most reliable serum biomarker for AD severity in a systematic review and meta-analysis, suggesting that it could potentially be a valuable biomarker for both assessing disease severity in AD and evaluating the course of disease [13]. TARC/CCL17 is a CC chemokine and member of the Th2 chemokine family and is involved in the recruitment of T cells into the skin [28,31]. A high correlation between AD severity and serum TARC/CCL17 levels has also been confirmed in pediatric patients [32,33,34,35,36]. The normal level of serum TARC/CCL17 is less than 450 pg/mL in healthy adults, while in healthy children its level varies depending on age [18,36]. However, TARC/CCL17 levels have been found to vary between AD patients with similar disease severity scores [37], while some patients with severe AD may occasionally have normal or low levels of serum TARC/CCL17, findings reflecting the heterogeneity of AD pathogenesis [13,18]. Since 2008 in Japan, serum TARC/CCL17 levels have been commercially measured under health insurance support and TARC/CCL17 has become a valuable clinical biomarker for monitoring response to treatment [18,38], as well.

Other biomarkers for AD severity include markers correlated with general inflammation or allergy such as C-reactive protein (CRP) [39], serum lactate dehydrogenase (LDH) [39,40,41], peripheral eosinophil count and serum eosinophil cationic protein (ECP), a protein released during the degranulation of eosinophils [13,40,41,42,43,44]. Regarding total serum IgE, although it has been the most studied biomarker in AD, it was demonstrated to only correlate weakly with AD severity during follow-up of patients [13,41,45,46]. In addition, although patients with severe disease tend to have elevated total serum IgE levels, in another group of patients, especially with intrinsic AD, IgE levels are not increased, implying that total serum IgE is not suitable as a biomarker for monitoring disease severity [45,47]. The ratio between specific IgE level against a particular allergen and total IgE level has been suggested to be a more useful AD biomarker [8].

Periostin, a matricellular protein that plays an important role in the pathogenesis of AD promoting the Th2 immune response [44,48], has also been suggested as a potential serum AD biomarker correlating with disease severity [44,48,49,50], although further investigation is needed. Moreover, in adult patients with AD, disease severity has been associated with *Staphylococcus aureus* skin colonization, resulting in more severe disease, barrier disruption, elevation of levels of type 2 biomarkers (eosinophil count, TARC/CCL17, IgE, periostin) and LDH, and greater allergen sensitization [51].

Regarding IL-31, a pruritogenic cytokine associated with atopic itch [52], and its use as a potential biomarker correlating with AD severity, the published data are currently controversial [53,54,55,56,57,58]. Additionally, potential biomarkers associated with skin barrier function, such as filaggrin (FLG) and natural moisturizing factor (NMF), may inversely correlate with AD severity [18,59].

### 3.2. Prognostic and Screening Biomarkers

The best-established risk factor for AD is a positive family history of atopic disease. Since it has been reported that the use of emollients may prevent the development of AD in high-risk infants [60,61], it might also be useful to distinguish prognostic and screening biomarkers that could identify and treat the population at risk [47].

The strongest genetic risk factor for AD barrier dysfunction is loss of function mutations in the FLG gene [62]. However, FLG gene mutations are not found in all AD patients, neither do all FLG mutation carriers develop AD [63,64]. FLG gene mutations have been associated with severe and early onset AD persisting into adulthood. Consequently, FLG mutations might be used as a prognostic and screening biomarker for severe or early onset disease [47,65]. Regarding AD comorbidities, low levels of FLG and high levels of IgE have been associated with the development of food allergy as part of the atopic march in patients with AD [18,66]. In addition, FLG breakdown products together with keratin 5 (KRT5), KRT14, KRT16 and AD clinical severity have been described as prognostic biomarkers for concomitant food allergy in children with AD [67].

Other suggested biomarkers which may predict the development of AD in infancy include elevated umbilical cord serum IgE and TARC/CCL17 levels [68,69], epidermal thymic stromal lymphopoietin protein (TSLP) expression [70], decreased level of natural moisturizing factor (NMF) and measurement of transepidermal water loss (TEWL) in newborns’ skin [71,72]. Moreover, a low serum vascular endothelial growth factor (VEGF) level has been found to predict AD persistence in infancy [73], while the enzyme indoleamine 2,3-dioxygenase-1 (IDO1) has been proposed as a prognostic candidate biomarker for the development of eczema herpeticum and other viral complications in AD patients [74].

Therefore, a combination of genetic factors and serum or tissue biomarkers might enhance the predictive value of potential prognostic biomarkers in AD [47].

### 3.3. Predictive Biomarkers

The multifactorial and complex pathophysiology of AD involving multiple immune pathways results in different levels of therapeutic response among AD patients. Therefore, the development of biomarkers predicting treatment response to a given therapy and particularly to a targeted therapy for AD is significantly important. Predictive biomarkers that identify the subpopulations of patients most likely to respond to a specific therapy may be common to all treatments (disease response biomarkers) or may be specific to an individual treatment (treatment-specific biomarkers) [18].

For instance, high serum periostin and dipeptidyl peptidase-4 (DPP-4) levels in AD patients have been reported as significant biomarkers to predict a good response to anti-IL-13 (tralokinumab) treatment [9]. High tissue IL-22 level at baseline has been identified as a potential treatment response biomarker for IL-22 inhibition (fezakinumab) [75]. Moreover, CXCL9 (Th1/interferon-related cytokine) and CXCL2 (Th17-related cytokine) have been suggested as treatment-specific predictive biomarkers for cyclosporine and dupilumab, respectively [76]. In addition, MDC/CCL22 has been proposed as a disease response biomarker regardless of the therapeutic modality used and its targeting pathway [76].

Furthermore, the inflammatory response and immunological profile of AD patients belonging to different age groups or ethnicities might also determine predictive biomarkers for treatment response concerning these subsets of patients. Although excess Th2 activation characterizes both children and adults with AD, high Th9 and Th17 activation has been demonstrated in children with AD [77], while higher levels of Th22 inflammation have been found in adults with AD [78]. Thus, it could be suggested that these age groups of patients would benefit from therapies targeting different cytokines according to the expressed inflammatory response [8]. Similarly, Asian patients exhibit higher Th17/Th22 activation [79,80], European American patients are characterized by increased Th2/Th22 and lower Th1/Th17 expression, while African American patients exhibit Th2/Th22 predominance and distinct Th1/Th17 attenuation [80]. As a result, the differential expression of inflammatory cytokines might have important implications on the response to treatment among different ethnic groups with AD [81].

### 3.4. Diagnostic Biomarkers

To date, the diagnosis of AD is exclusively based on clinical criteria and established biomarkers to confirm the diagnosis are lacking.

In special cases, AD phenotypes may overlap with psoriasis causing differential diagnostic problems. Therefore, potential diagnostic biomarkers have been proposed to improve diagnosis by differentiating AD and psoriasis in patients with psoriasiform dermatitis, namely nitric oxide synthase 2 (NOS2) and CTACK/CCL27 [82,83,84].

**Figure 1:**
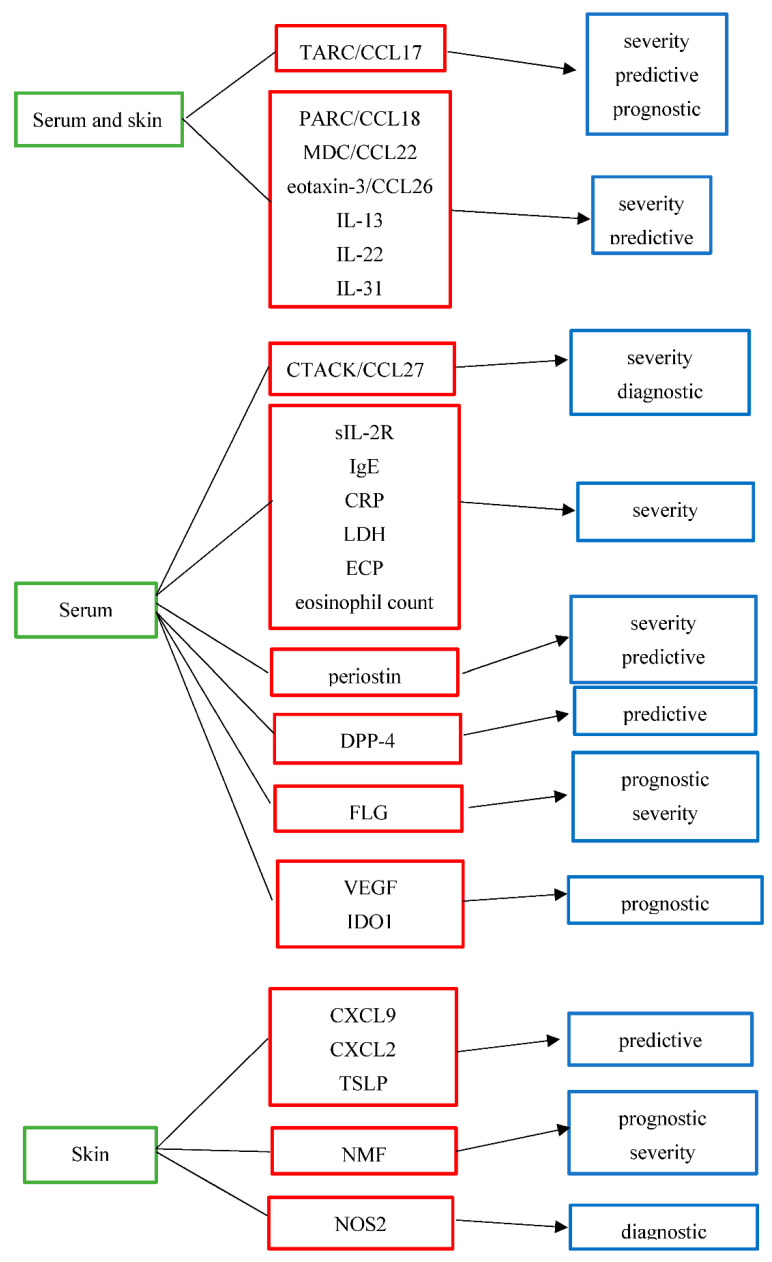
Biologic origin and suggested use of potential biomarkers in atopic dermatitis. TARC/CCL17: thymus and activation-regulated chemokine/C-C motif ligand 17, PARC/CCL18: pulmonary and activation-regulated chemokine/C-C motif ligand 18, MDC/CCL22: macrophage-derived chemokine/C-C motif ligand 22, eotaxin-3/CCL26: eosinophil-attracting chemokine/C-C motif ligand 26, IL-13:interleukin-13, IL-22:interleukin-22, IL-31:interleukin-31, CTACK/CCL27:cutaneous T-cell-attracting chemokine/C-C motif ligand 27, sIL-2R:soluble IL-2 receptor, IgE:immunoglobulin E, CRP:C-reactive protein, LDH:lactate dehydrogenase, ECP:eosinophil cationic protein, DPP-4:dipeptidyl peptidase-4, FLG:filaggrin, VEGF:vascular endothelial growth factor, IDO1:indoleamine 2,3-dioxygenase-1, CXCL9:C-X-C motif ligand 9, CXCL2:C-X-C motif ligand 2,TSLP: thymic stromal lymphopoietin protein, NMF:natural moisturizing factor, NOS2:nitric oxide synthase 2.

The above-mentioned candidate biomarkers for AD and their suggested use are summarized in Table 1 and Figure 1.

## 4. Conclusions

AD is an immune-driven disease with dramatic impact on patients’ and their families’ qualities of life, representing a significant socioeconomic burden. Recently it has become clear that AD is a highly heterogeneous disease regarding its clinical presentations, course, degree and type of underlying inflammation, and response to therapy. However, to date the management of AD does not take into consideration the multiple phenotypes and endotypes of the disease leading to an unmet medical need for effective and personalized treatment. To this end, biomarkers are expected to contribute to the better identification and stratification of patients with AD according to their molecular and clinical characteristics, resulting in patient-tailored therapy.

Since scientific justification and interpretation of biomarkers are not always reliable and accurate, one principal challenge is to distinguish between a potential biomarker and a reliable biomarker that can be universally used to guide important clinical and commercial decisions [19]. Moreover, the development of a new biomarker from discovery to validation, qualification, and clinical use is a rather complex and demanding procedure, often comparable to a drug development process [18].

It should be emphasized that despite extended clinical research and identification of several potential biomarkers in AD, to date, none of these candidate biomarkers has been validated and officially accepted by regulatory organizations for use in routine clinical practice and management of AD. Among the potential biomarkers in AD that have been identified so far, TARC/CCL17 has accumulated the greatest evidence to become a reliable AD biomarker strongly correlated with disease severity in both children and adults. In addition, the most prominent candidate AD biomarkers include Th2-related chemokines namely MDC/CCL22, PARC/CCL18, CTACK/CCL27, eotaxin-3/CCL26, the key Th2 cytokine IL-13 and the key Th22 cytokine IL-22 [18], although further investigation is needed.

During the last few years, AD has experienced a revolution in the field of treatment with the development of novel targeted, highly specific, but expensive therapies, emphasizing the need for a precision medicine approach. The implementation of precision medicine in patients with AD requires the identification and validation of objective and reliable biomarkers that may distinguish which patients are most likely to benefit from specific targeted therapies.

To be useful in clinical practice, these biomarkers should be accurate, reproducible, minimally invasive, clinically applicable, and easily measured. Furthermore, regarding their role in the management of AD, reliable biomarkers should improve diagnosis, gauge disease severity, or predict the course of disease, the development of comorbidities, or the therapeutic response, resulting in optimized and personalized treatment in AD. Finally, in a complex disease such as AD, combined biomarkers may prove more reliable compared to the use of an individual biomarker, but additional research is required.

## Figures and Tables

**Table 1:** Candidate biomarkers in atopic dermatitis.

Biomarker	Full Name	Purpose	Grade of Evidence	Biologic Origin
TARC/CCL17 [13,25,27,28,29,30,32,33,34,35,36,37,38,40,53,69]	thymus and activation-regulated chemokine/ C-C motif ligand 17	severity predictive prognostic	high moderate low	serum and skin
PARC/CCL18 [25,27,28,29]	pulmonary and activation-regulated chemokine/C-C motif ligand 18	severity predictive	moderate low	serum and skin
MDC/CCL22 [13,25,27,28,29,30,76]	macrophage-derived chemokine/C-C motif ligand 22	severity predictive	moderate low	serum and skin
Eotaxin-3/CCL26 [25,27]	eosinophil-attracting chemokine/C-C motif ligand 26	severity predictive	moderate low	serum and skin
IL-13 [9,25,27,77,78,79,80]	interleukin-13	severity predictive	moderate low	serum and skin
IL-22 [25,27,28,29,75,77,78,79,80]	interleukin-22	severity predictive	moderate low	serum and skin
IL-31 [13,52,53,54,55,56,57,58]	interleukin-31	severity predictive	moderate low	serum and skin
CTACK/CCL27 [13,25,27,33,34,82,83,84]	cutaneous T-cell-attracting chemokine/ C-C motif ligand 27	severity diagnostic/differential diagnosis from psoriasis	moderate low	serum
sIL-2R [13,28,29]	soluble IL-2 receptor	severity	moderate	serum
IgE [13,40,41,45,46]	immunoglobulin E	severity	moderate	serum
CRP [39]	C-reactive protein	severity	low	serum
LDH [13,39,40,41]	lactate dehydrogenase	severity	moderate	serum
ECP [13,42,43]	eosinophil cationic protein	severity	moderate	serum
Eosinophil count [40,41,43,44]		severity	moderate	serum
Periostin [9,44,48,49,50]		severity predictive	moderate low	serum
DPP-4 [9]	dipeptidyl peptidase-4	predictive	low	serum
CXCL9 [76]	C-X-C motif ligand 9	predictive	low	skin
CXCL2 [76]	C-X-C motif ligand 2	predictive	low	skin
TSLP [70]	thymic stromal lymphopoietin protein	predictive	moderate	skin
NMF [59,71]	natural moisturizing factor	prognostic severity	moderate low	skin
FLG [47,59,62,65,66,67]	filaggrin	prognostic severity	moderate low	serum
VEGF [73]	vascular endothelial growth factor	prognostic	low	serum
IDO1 [74]	indoleamine 2,3-dioxygenase-1	prognostic for eczema herpeticum	low	serum
NOS2 [82,83,84]	nitric oxide synthase 2	diagnostic/differential diagnosis from psoriasis	low	skin

## References

[1] Eichenfield L.F., Tom W.L., Chamlin S.L., Feldman S.R., Hanifin J.M., Simpson E.L., Berger T.G., Bergman J.N., Cohen D.E., Cooper K.D. (2014). Guidelines of care for the management of atopic dermatitis: Section 1. Diagnosis and assessment of atopic dermatitis. J. Am. Acad. Dermatol..

[2] Ziehfreund S., Tizek L., Hangel N., Fritzsche M., Weidinger S., Smith C., Bryce P., Greco D., Bogaard E., Flohr C. (2022). Requirements and expectations of high-quality biomarkers for atopic dermatitis and psoriasis in 2021—A two-round Delphi survey among international experts. J. Eur. Acad. Dermatol. Venereol..

[3] Moyle M., Cevikbas F., Harden J.L., Guttman-Yassky E. (2019). Understanding the immune landscape in atopic dermatitis: The era of biologics and emerging therapeutic approaches. Exp. Dermatol..

[4] Werfel T., Allam J.-P., Biedermann T., Eyerich K., Gilles S., Guttman-Yassky E., Hoetzenecker W., Knol E., Simon H.-U., Wollenberg A. (2016). Cellular and molecular immunologic mechanisms in patients with atopic dermatitis. J. Allergy Clin. Immunol..

[5] Bakker D.S., Nierkens S., Knol E.F., Giovannone B., Delemarre E.M., van der Schaft J., van Wijk F., de Bruin-Weller M.S., Drylewicz J., Thijs J.L. (2021). Confirmation of multiple endotypes in atopic dermatitis based on serum biomarkers. J. Allergy Clin. Immunol..

[6] Brunner P.M., Guttman-Yassky E., Leung D.Y. (2017). The immunology of atopic dermatitis and its reversibility with broad-spectrum and targeted therapies. J. Allergy Clin. Immunol..

[7] Mansouri Y., Guttman-Yassky E. (2015). Immune Pathways in Atopic Dermatitis, and Definition of Biomarkers through Broad and Targeted Therapeutics. J. Clin. Med..

[8] Bieber T., D’Erme A.M., Akdis C.A., Traidl-Hoffmann C., Lauener R., Schäppi G., Schmid-Grendelmeier P. (2017). Clinical phenotypes and endophenotypes of atopic dermatitis: Where are we, and where should we go?. J. Allergy Clin. Immunol..

[9] Wollenberg A., Howell M.D., Guttman-Yassky E., Silverberg J.I., Kell C., Ranade K., Moate R., van der Merwe R. (2019). Treatment of atopic dermatitis with tralokinumab, an anti–IL-13 mAb. J. Allergy Clin. Immunol..

[10] Han Y., Chen Y., Liu X., Zhang J., Su H., Wen H., Li W., Yao X. (2017). Efficacy and safety of dupilumab for the treatment of adult atopic dermatitis: A meta-analysis of randomized clinical trials. J. Allergy Clin. Immunol..

[11] Simpson E.L., Flohr C., Eichenfield L.F., Bieber T., Sofen H., Taïeb A., Owen R., Putnam W., Castro M., DeBusk K. (2018). Efficacy and safety of lebrikizumab (an anti-IL-13 monoclonal antibody) in adults with moderate-to-severe atopic dermatitis inadequately controlled by topical corticosteroids: A randomized, placebo-controlled phase II trial (TREBLE). J. Am. Acad. Dermatol..

[12] Guttman-Yassky E., Silverberg J.I., Nemoto O., Forman S.B., Wilke A., Prescilla R., de la Peña A., Nunes F.P., Janes J., Gamalo M. (2019). Baricitinib in adult patients with moderate-to-severe atopic dermatitis: A phase 2 parallel, double-blinded, randomized placebo-controlled multiple-dose study. J. Am. Acad. Dermatol..

[13] Thijs J., Krastev T., Weidinger S., Buckens C.F., De Bruin-Weller M., Bruijnzeel-Koomen C., Flohr C., Hijnen D. (2015). Biomarkers for atopic dermatitis: A systematic review and meta-analysis. Curr. Opin. Allergy Clin. Immunol..

[14] Biomarkers Definitions Working Group (2001). Biomarkers and surrogate endpoints: Preferred definitions and conceptual framework. Clin. Pharmacol. Ther..

[15] (2001). WHO International Programme on Chemical Safety Biomarkers in Risk Assessment: Validity and Validation. http://www.inchem.org/documents/ehc/ehc/ehc222.htm.

[16] FDA-NIH Biomarker Working Group (2016). BEST (Biomarkers, EndpointS, and Other Tools) Resource. https://www.ncbi.nlm.nih.gov/books/NBK326791/.

[17] EMA (2020). Glossary: Biomarker. https://www.ema.europa.eu/en/glossary/biomarker.

[18] Renert-Yuval Y., Thyssen J.P., Bissonnette R., Bieber T., Kabashima K., Hijnen D., Guttman-Yassky E. (2021). Biomarkers in atopic dermatitis—a review on behalf of the International Eczema Council. J. Allergy Clin. Immunol..

[19] Gromova M., Vaggelas A., Dallmann G., Seimetz D. (2020). Biomarkers: Opportunities and Challenges for Drug Development in the Current Regulatory Landscape. Biomark. Insights.

[20] Guttman-Yassky E., Diaz A., Pavel A.B., Fernandes M., Lefferdink R., Erickson T., Canter T., Rangel S., Peng X., Li R. (2019). Use of Tape Strips to Detect Immune and Barrier Abnormalities in the Skin of Children With Early-Onset Atopic Dermatitis. JAMA Dermatol..

[21] Castelo-Soccio L. (2019). Stripping Away Barriers to Find Relevant Skin Biomarkers for Pediatric Atopic Dermatitis. JAMA Dermatol..

[22] Pavel A.B., Renert-Yuval Y., Wu J., Del Duca E., Diaz A., Lefferdink R., Fang M.M., Canter T., Rangel S.M., Zhang N. (2020). Tape strips from early-onset pediatric atopic dermatitis highlight disease abnormalities in nonlesional skin. Allergy.

[23] Kim B.E., Goleva E., Kim P.S., Norquest K., Bronchick C., Taylor P., Leung D.Y. (2019). Side-by-Side Comparison of Skin Biopsies and Skin Tape Stripping Highlights Abnormal Stratum Corneum in Atopic Dermatitis. J. Investig. Dermatol..

[24] Thijs J.L., Fiechter R., Giovannone B., de Bruin-Weller M.S., Knol E.F., Bruijnzeel-Koomen C.A.F.M., Drylewicz J., Nierkens S., Hijnen D. (2019). Biomarkers detected in dried blood spots from atopic dermatitis patients strongly correlate with disease severity. Allergy.

[25] Ungar B., Garcet S., Gonzalez J., Dhingra N., da Rosa J.C., Shemer A., Krueger J.G., Suarez-Farinas M., Guttman-Yassky E. (2017). An Integrated Model of Atopic Dermatitis Biomarkers Highlights the Systemic Nature of the Disease. J. Investig. Dermatol..

[26] Thijs J.L., Van Seggelen W., Bruijnzeel-Koomen C., De Bruin-Weller M., Hijnen D. (2015). New Developments in Biomarkers for Atopic Dermatitis. J. Clin. Med..

[27] He H., Del Duca E., Diaz A., Kim H.J., Gay-Mimbrera J., Zhang N., Wu J., Beaziz J., Estrada Y., Krueger J.G. (2021). Mild atopic dermatitis lacks systemic inflammation and shows reduced nonlesional skin abnormalities. J. Allergy Clin. Immunol..

[28] Thijs J.L., Nierkens S., Herath A., Bruijnzeel-Koomen C.A., Knol E., Giovannone B., De Bruin-Weller M.S., Hijnen D. (2015). A panel of biomarkers for disease severity in atopic dermatitis. Clin. Exp. Allergy.

[29] Thijs J.L., Drylewicz J., Fiechter R., Strickland I., Sleeman M.A., Herath A., May R.D., Bruijnzeel-Koomen C.A., Knol E.F., Giovannone B. (2017). EASI p-EASI: Utilizing a combination of serum biomarkers offers an objective measurement tool for disease severity in atopic dermatitis patients. J. Allergy Clin. Immunol..

[30] Hulshof L., Overbeek S.A., Wyllie A.L., Chu M.L.J.N., Bogaert D., De Jager W., Knippels L.M.J., Sanders E.A.M., Van Aalderen W.M.C., Garssen J. (2018). Exploring Immune Development in Infants With Moderate to Severe Atopic Dermatitis. Front Immunol. Front. Immunol..

[31] Imai T., Yoshida T., Baba M., Nishimura M., Kakizaki M., Yoshie O. (1996). Molecular Cloning of a Novel T Cell-directed CC Chemokine Expressed in Thymus by Signal Sequence Trap Using Epstein-Barr Virus Vector. J. Biol. Chem..

[32] Song T.W., Sohn M.H., Kim E.S., Kim K.W., Kim K.-E. (2006). Increased serum thymus and activation-regulated chemokine and cutaneous T cell-attracting chemokine levels in children with atopic dermatitis. Clin. Exp. Allergy.

[33] Hijnen D., De Bruin-Weller M., Oosting B., Lebre C., De Jong E., Bruijnzeel-Koomen C., Knol E. (2004). Serum thymus and activation-regulated chemokine (TARC) and cutaneous T cell–attracting chemokine (CTACK) levels in allergic diseases: TARC and CTACK are disease-specific markers for atopic dermatitis. J. Allergy Clin. Immunol..

[34] Machura E., Rusek-Zychma M., Jachimowicz M., Wrzask M., Mazur B., Kasperska-Zajac A. (2012). Serum TARC and CTACK concentrations in children with atopic dermatitis, allergic asthma, and urticaria. Pediatr. Allergy Immunol..

[35] Ahrens B., Schulz G., Bellach J., Niggemann B., Beyer K. (2015). Chemokine levels in serum of children with atopic dermatitis with regard to severity and sensitization status. Pediatr. Allergy Immunol..

[36] Fujisawa T., Nagao M., Hiraguchi Y., Katsumata H., Nishimori H., Iguchi K., Kato Y., Higashiura M., Ogawauchi I., Tamaki K. (2009). Serum measurement of thymus and activation-regulated chemokine/CCL17 in children with atopic dermatitis: Elevated normal levels in infancy and age-specific analysis in atopic dermatitis. Pediatr. Allergy Immunol..

[37] Landheer J., de Bruin-Weller M., Boonacker C., Hijnen D., Bruijnzeel-Koomen C., Röckmann H. (2014). Utility of serum thymus and activation-regulated chemokine as a biomarker for monitoring of atopic dermatitis severity. J. Am. Acad. Dermatol..

[38] Yasukochi Y., Nakahara T., Abe T., Kido-Nakahara M., Kohda F., Takeuchi S., Hagihara A., Furue M. (2014). Reduction of serum TARC levels in atopic dermatitis by topical anti-inflammatory treatments. Asian Pac. J. Allergy Immunol..

[39] Vekaria A.S., Brunner P.M., Aleisa A.I., Bonomo L., Lebwohl M.G., Israel A., Guttman-Yassky E. (2017). Moderate-to-severe atopic dermatitis patients show increases in serum C-reactive protein levels, correlating with skin disease activity. F1000Research.

[40] Kou K., Aihara M., Matsunaga T., Chen H., Taguri M., Morita S., Fujita H., Yamaguchi Y., Kambara T., Ikezawa Z. (2012). Association of serum interleukin-18 and other biomarkers with disease severity in adults with atopic dermatitis. Arch. Dermatol. Res..

[41] Morishima Y., Kawashima H., Takekuma K., Hoshika A. (2010). Changes in serum lactate dehydrogenase activity in children with atopic dermatitis. Pediatr. Int..

[42] Czech W., Krutmann J., Schopf E., Kapp A. (1992). Serum eosinophil cationic protein (ECP) is a sensitive measure for disease activity in atopic dermatitis. Br. J. Dermatol..

[43] Kägi M.K., Joller-Jemelka H., Wüthrich B. (1992). Correlation of Eosinophils, Eosinophil Cationic Protein and Soluble lnterleukin-2 Receptor with the Clinical Activity of Atopic Dermatitis. Dermatology.

[44] Kou K., Okawa T., Yamaguchi Y., Ono J., Inoue Y., Kohno M., Matsukura S., Kambara T., Ohta S., Izuhara K. (2014). Periostin levels correlate with disease severity and chronicity in patients with atopic dermatitis. Br. J. Dermatol..

[45] Thijs J.L., Knipping K., Bruijnzeel-Koomen C.A.F., Garssen J., De Bruin-Weller M.S., Hijnen D.J. (2016). Immunoglobulin free light chains in adult atopic dermatitis patients do not correlate with disease severity. Clin. Transl. Allergy.

[46] Aral M., Arican O., Gül M., Sasmaz S., Kocturk S.A., Kastal U., Ekerbicer H.C. (2006). The relationship between serum levels of total IgE, IL-18, IL-12, IFN-gamma and disease severity in children with atopic dermatitis. Mediat. Inflamm..

[47] Thijs J.L., de Bruin-Weller M.S., Hijnen D. (2017). Current and Future Biomarkers in Atopic Dermatitis. Immunol. Allergy Clin. N. Am..

[48] Masuoka M., Shiraishi H., Ohta S., Suzuki S., Arima K., Aoki S., Toda S., Inagaki N., Kurihara Y., Hayashida S. (2012). Periostin promotes chronic allergic inflammation in response to Th2 cytokines. J. Clin. Investig..

[49] Yamaguchi Y. (2014). Periostin in skin tissue and skin-related diseases. Allergol. Int..

[50] Sung M., Lee K.S., Ha E.G., Lee S.J., Kim M.A., Lee S.W., Jee H.M., Sheen Y.H., Jung Y.H., Han M.Y. (2017). An association of periostin levels with the severity and chronicity of atopic dermatitis in children. Pediatr. Allergy Immunol..

[51] Simpson E.L., Villarreal M., Jepson B., Rafaels N., David G., Hanifin J., Taylor P., Boguniewicz M., Yoshida T., De Benedetto A. (2018). Patients with Atopic Dermatitis Colonized with Staphylococcus aureus Have a Distinct Phenotype and Endotype. J. Investig. Dermatol..

[52] Furue M., Yamamura K., Kido-Nakahara M., Nakahara T., Fukui Y. (2018). Emerging role of interleukin-31 and interleukin-31 receptor in pruritus in atopic dermatitis. Allergy.

[53] Kyoya M., Kawakami T., Soma Y. (2014). Serum thymus and activation-regulated chemokine (TARC) and interleukin-31 levels as biomarkers for monitoring in adult atopic dermatitis. J. Dermatol. Sci..

[54] Raap U., Weißmantel S., Gehring M., Eisenberg A.M., Kapp A., Fölster-Holst R. (2012). IL-31 significantly correlates with disease activity and Th2 cytokine levels in children with atopic dermatitis. Pediatr. Allergy Immunol..

[55] Neis M.M., Peters B., Dreuw A., Wenzel J., Bieber T., Mauch C., Krieg T., Stanzel S., Heinrich P.C., Merk H.F. (2006). Enhanced expression levels of IL-31 correlate with IL-4 and IL-13 in atopic and allergic contact dermatitis. J. Allergy Clin. Immunol..

[56] Raap U., Wichmann K., Bruder M., Ständer S., Wedi B., Kapp A., Werfel T. (2008). Correlation of IL-31 serum levels with severity of atopic dermatitis. J. Allergy Clin. Immunol..

[57] Nygaard U., Hvid M., Johansen C., Buchner M., Fölster-Holst R., Deleuran M., Vestergaard C. (2016). TSLP, IL-31, IL-33 and sST2 are new biomarkers in endophenotypic profiling of adult and childhood atopic dermatitis. J. Eur. Acad. Dermatol. Venereol..

[58] Ozceker D., Bulut M., Ozbay A.C., Dilek F., Koser M., Tamay Z., Guler N. (2018). Assessment of IL-31 levels and disease severity in children with atopic dermatitis. Allergol. Immunopathol..

[59] Kezic S., O’Regan G.M., Lutter R., Jakasa I., Koster E.S., Saunders S., Caspers P., Kemperman P.M., Puppels G.J., Sandilands A. (2012). Filaggrin loss-of-function mutations are associated with enhanced expression of IL-1 cytokines in the stratum corneum of patients with atopic dermatitis and in a murine model of filaggrin deficiency. J. Allergy Clin. Immunol..

[60] Simpson E.L., Chalmers J.R., Hanifin J.M., Thomas K.S., Cork M.J., McLean W.I., Brown S.J., Chen Z., Chen Y., Williams H.C. (2014). Emollient enhancement of the skin barrier from birth offers effective atopic dermatitis prevention. J. Allergy Clin. Immunol..

[61] Glatz M., Jo J.-H., Kennedy E.A., Polley E.C., Segre J.A., Simpson E.L., Kong H.H. (2018). Emollient use alters skin barrier and microbes in infants at risk for developing atopic dermatitis. PLoS ONE.

[62] Paternoster L., Standl M., Waage J., Baurecht H., Hotze M., Strachan D.P., Curtin J.A., Bønnelykke K., Tian C., Takahashi A. (2015). Multi-ancestry genome-wide association study of 21,000 cases and 95,000 controls identifies new risk loci for atopic dermatitis. Nat. Genet..

[63] Kezic S., O’Regan G.M., Yau N., Sandilands A., Chen H., Campbell L.E., Kroboth K., Watson R., Rowland M., Irwin McLean W.H. (2011). Levels of filaggrin degradation products are influenced by both filaggrin genotype and atopic dermatitis severity. Allergy.

[64] Weidinger S., O’Sullivan M., Illig T., Baurecht H., Depner M., Rodriguez E., Ruether A., Klopp N., Vogelberg C., Weiland S.K. (2008). Filaggrin mutations, atopic eczema, hay fever, and asthma in children. J. Allergy Clin. Immunol..

[65] Barker J.N., Palmer C.N., Zhao Y., Liao H., Hull P.R., Lee S.P., Allen M.H., Meggitt S.J., Reynolds N.J., Trembath R.C. (2007). Null Mutations in the Filaggrin Gene (FLG) Determine Major Susceptibility to Early-Onset Atopic Dermatitis that Persists into Adulthood. J. Investig. Dermatol..

[66] Tham E.H., Leung D.Y. (2019). Mechanisms by Which Atopic Dermatitis Predisposes to Food Allergy and the Atopic March. Allergy Asthma Immunol. Res..

[67] Leung D.Y.M., Calatroni A., Zaramela L.S., LeBeau P.K., Dyjack N., Brar K., David G., Johnson K., Leung S., Ramirez-Gama M. (2019). The nonlesional skin surface distinguishes atopic dermatitis with food allergy as a unique endotype. Sci. Transl. Med..

[68] Wen H.-J., Wang Y.-J., Lin Y.-C., Chang C.-C., Shieh C.-C., Lung F.-W., Guo Y.L. (2011). Prediction of atopic dermatitis in 2-yr-old children by cord blood IgE, genetic polymorphisms in cytokine genes, and maternal mentality during pregnancy. Pediatr. Allergy Immunol..

[69] Miyahara H., Okazaki N., Nagakura T., Korematsu S., Izumi T. (2011). Elevated umbilical cord serum TARC/CCL17 levels predict the development of atopic dermatitis in infancy. Clin. Exp. Allergy.

[70] Kim J., Kim B.E., Lee J., Han Y., Jun H.-Y., Kim H., Choi J., Leung D.Y., Ahn K. (2016). Epidermal thymic stromal lymphopoietin predicts the development of atopic dermatitis during infancy. J. Allergy Clin. Immunol..

[71] Ní Chaoimh C., Nico C., Puppels G.J., Caspers P.J., Wong X.F.C.C., Common J.E., Irvine A.D., Hourihane J.O. (2020). In vivo Raman spectroscopy discriminates between FLG loss-of-function carriers vs. wild-type in day 1-4 neonates. Ann. Allergy, Asthma Immunol..

[72] Horimukai K., Morita K., Narita M., Kondo M., Kabashima S., Inoue E., Sasaki T., Niizeki H., Saito H., Matsumoto K. (2015). Transepidermal water loss measurement during infancy can predict the subsequent development of atopic dermatitis regardless of filaggrin mutations. Allergol. Int..

[73] Lauffer F., Baghin V., Standl M., Stark S.P., Jargosch M., Wehrle J., Thomas J., Schmidt-Weber C.B., Biedermann T., Eyerich S. (2021). Predicting persistence of atopic dermatitis in children using clinical attributes and serum proteins. Allergy.

[74] Staudacher A., Hinz T., Novak N., Von Bubnoff D., Bieber T. (2015). Exaggerated IDO1 expression and activity in Langerhans cells from patients with atopic dermatitis upon viral stimulation: A potential predictive biomarker for high risk of Eczema herpeticum. Allergy.

[75] Brunner P.M., Pavel A.B., Khattri S., Leonard A., Malik K., Rose S., On S.J., Vekaria A.S., Traidl-Hoffmann C., Singer G.K. (2018). Baseline IL-22 expression in patients with atopic dermatitis stratifies tissue responses to fezakinumab. J. Allergy Clin. Immunol..

[76] Glickman J.W., Han J., Garcet S., Krueger J.G., Pavel A.B., Guttman-Yassky E. (2020). Improving evaluation of drugs in atopic dermatitis by combining clinical and molecular measures. J. Allergy Clin. Immunol. Pract..

[77] Esaki H., Brunner P.M., Renert-Yuval Y., Czarnowicki T., Huynh T., Tran G., Lyon S., Rodriguez G., Immaneni S., Johnson D.B. (2016). Early-onset pediatric atopic dermatitis is T H 2 but also T H 17 polarized in skin. J. Allergy Clin. Immunol..

[78] Czarnowicki T., Esaki H., Gonzalez J., Malajian D., Shemer A., Noda S., Talasila S., Berry A., Gray J., Becker L. (2015). Early pediatric atopic dermatitis shows only a cutaneous lymphocyte antigen (CLA)+ TH2/TH1 cell imbalance, whereas adults acquire CLA+ TH22/TC22 cell subsets. J. Allergy Clin. Immunol..

[79] Noda S., Suárez-Fariñas M., Ungar B., Kim S.J., de Guzman Strong C., Xu H., Peng X., Estrada Y.D., Nakajima S., Honda T. (2015). The Asian atopic dermatitis phenotype combines features of atopic dermatitis and psoriasis with increased TH17 polarization. J. Allergy Clin. Immunol..

[80] Nomura T., Wu J., Kabashima K., Guttman-Yassky E. (2020). Endophenotypic Variations of Atopic Dermatitis by Age, Race, and Ethnicity. J. Allergy Clin. Immunol. Pract..

[81] Kaufman B.P., Guttman-Yassky E., Alexis A.F. (2018). Atopic dermatitis in diverse racial and ethnic groups-Variations in epidemiology, genetics, clinical presentation and treatment. Exp. Dermatol..

[82] Quaranta M., Knapp B., Garzorz N., Mattii M., Pullabhatla V., Pennino D., Andres C., Traidl-Hoffmann C., Cavani A., Theis F.J. (2014). Intraindividual genome expression analysis reveals a specific molecular signature of psoriasis and eczema. Sci. Transl. Med..

[83] Garzorz-Stark N., Krause L., Lauffer F., Atenhan A., Thomas J., Stark S.P., Franz R., Weidinger S., Balato A., Mueller N.S. (2016). A novel molecular disease classifier for psoriasis and eczema. Exp. Dermatol..

[84] He H., Bissonnette R., Wu J., Diaz A., Proulx E.S.-C., Maari C., Jack C., Louis M., Estrada Y., Krueger J.G. (2021). Tape strips detect distinct immune and barrier profiles in atopic dermatitis and psoriasis. J. Allergy Clin. Immunol..

